# Structural plasticity of olfactory neuropils in relation to insect diapause

**DOI:** 10.1002/ece3.7046

**Published:** 2020-11-18

**Authors:** Maertha Eriksson, Niklas Janz, Sören Nylin, Mikael A. Carlsson

**Affiliations:** ^1^ Department of Zoology Stockholm University Stockholm Sweden

**Keywords:** antennal lobe, brain, butterfly, diapause, insect, mushroom body, olfaction, plasticity

## Abstract

1. Many insects that live in temperate zones spend the cold season in a state of dormancy, referred to as diapause. As the insect must rely on resources that were gathered before entering diapause, keeping a low metabolic rate is of utmost importance. Organs that are metabolically expensive to maintain, such as the brain, can therefore become a liability to survival if they are too large.
2. Insects that go through diapause as adults generally do so before entering the season of reproduction. This order of events introduces a conflict between maintaining low metabolism during dormancy and emerging afterward with highly developed sensory systems that improve fitness during the mating season.
3. We investigated the timing of when investments into the olfactory system are made by measuring the volumes of primary and secondary olfactory neuropils in the brain as they fluctuate in size throughout the extended diapause life‐period of adult *Polygonia c‐album* butterflies.
4. Relative volumes of both olfactory neuropils increase significantly during early adult development, indicating the importance of olfaction to this species, but still remain considerably smaller than those of nondiapausing conspecifics. However, despite butterflies being kept under the same conditions as before the dormancy, their olfactory neuropil volumes decreased significantly during the postdormancy period.
5. The opposing directions of change in relative neuropil volumes before and after diapause dormancy indicate that the investment strategies governing structural plasticity during the two life stages could be functionally distinct. As butterflies were kept in stimulus‐poor conditions, we find it likely that investments into these brain regions rely on experience‐*expectant* processes before diapause and experience‐*dependent* processes after diapause conditions are broken.
6. As the shift in investment strategies coincides with a hard shift from premating season to mating season, we argue that these developmental characteristics could be adaptations that mitigate the trade‐off between dormancy survival and reproductive fitness.

## Graphical abstract

The structural plasticity of olfactory neuropils in butterflies going through adult diapause appears to be regulated by different mechanisms before and after the diapause event, and these neuropils are proportionally smaller in diapause‐ready butterflies compared with non‐diapausing conspecifics. As neural tissue is metabolically expensive to use and maintain, and since the diapause period in part is characterized by starvation, we argue that these developmental characteristics could be adaptations that mitigate a trade‐off between dormancy survival and reproductive success.

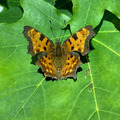

## 1 INTRODUCTION

As environmental resources in natural habitats are limited, resource management is one of the core concepts around which all survival strategies revolve. The task of balancing metabolic energy consumption to resource availability becomes increasingly challenging in environments where resource distribution is highly heterogeneous. In many areas of the world, resource abundancy is tightly connected to climatic seasonality, and even though the cyclic nature of seasons means high predictability of when and where resources will be available, temporal patchiness in resource abundance can lead to long periods of time when resource availability is extremely low. In order to survive prolonged periods of extreme resource scarcity without suffering detrimental effects, organisms must rely on genetically preprogrammed strategies for energy‐efficient long‐term resource allocation. The nature and details of these allocation strategies are vital to our understanding of how organisms have become adapted to their historical habitats, and how they may adapt to climatic changes in the future.

Many organisms living in habitats characterized by predictable cyclicity in resource availability have the ability to enter a state of dormancy (Hand et al., 2016). The dormancy functions as an escape mechanism for organisms, which cannot logistically avoid temporary adverse conditions, and may last anywhere from a few months to several years depending on species and habitat type (Gill et al., 2017). Among insects, a specialized version of seasonally induced dormancy, referred to as diapause, is commonly observed throughout a variety of habitat types and climatic zones (e.g., Denlinger, 1986; Diniz et al., 2017; Kosumi & Takeda, 2017; Pires et al., 2000; Sahoo et al., 2018; Santos et al., 2018; Wolda & Denlinger, 1984). In some species, diapause is facultatively induced by external cues, allowing the species to be multivoltine in suitable habitats, while other species are obligate diapausers and always have a single generation per season (Denlinger et al., 2012; Dolezel, 2015). The precise life history‐related details vary greatly between species, but the diapause state is generally characterized by slowed down or temporarily arrested ontogenetic development, low mobility, and low metabolic rate (Danks, 1987; Denlinger, 2002).

Despite often being referred to as a period of developmental arrest, diapause should be understood in the context of alternative developmental pathways rather than as being a static state or stagnant latency period (Koštál, 2006; Lehmann et al., 2018; Ragland et al., 2010). It includes several distinct developmental phases and is generally considered to be a metabolically expensive life history strategy, especially when experienced during mobile life stages (Hahn & Denlinger, 2007; Lehmann et al., 2016). The main part of the diapause‐related dormancy period of most insects consists of an endogenously regulated phase called diapause maintenance, where the animal is unresponsive to external stimuli, and an exogenously regulated phase called postdiapause quiescence (Koštál, 2006). While the exact conditions determining induction and termination of diapause may differ even among closely related species (Wiklund et al., 2019), the maintenance and postdiapause quiescence periods are likely to share many functional characteristics across genera (Denlinger, 2002; Denlinger et al., 1980; Ragland et al., 2010).

The internal processes and external triggers involved with diapause regulation are relatively well understood (Denlinger, 2002; Dolezel, 2015; Lehmann et al., 2018; Ragland et al., 2010; Saunders, 2014; Tougeron, 2019; Xu et al., 2012), but comparatively little is known about the state of sensory systems during this period. Considering that neural tissues are energetically expensive to use (Niven & Laughlin, 2008), one would expect the processes involved with nonessential sensory activity to be substantially down‐regulated during the diapause dormancy period. Consistent with this hypothesis, previous records of blowflies show decreased responsiveness to gustatory stimulation during diapause (Stoffolano, 1975). The costs associated with maintenance of neural tissues could likewise be expected to favor smaller brains that require less resources for up‐keep during this time. Alternatively, brain regions could display different growth patterns during different phases of the diapause dormancy period, concordant with the animal's needs at each phase (Lehmann et al., 2017). As metabolic suppression generally is less deep during adult diapause than it is during diapause at more stationary life stages (Koštál, 2006), reductions in metabolic costs for sensory systems could be especially important for adult diapausers. But as diapause generally is endured prior to reproductive events, adults must also make investments into development and maturation in order for the physiology to be optimized at the peak of the reproductive season. The contradictory nature of these two expectations leads back to the topic of strategic resource allocation, extending over a timescale that includes the periods both before and after the diapause event itself. Among the questions we may ask, one of the most fundamental queries involves the timing of investments into sensory systems in relation to the diapause dormancy period and mating season. Are investments into brain regions involved with sensory processing best suited to be made before, during, or after the diapause dormancy period?

A sensory system of particular importance to many insects is the one dedicated to olfactory information. Olfaction is an essential tool for locating not only food and potential mates, but also high‐quality oviposition sites (Hansson, 1999) which allow offspring an advantage during early life. Odorant information from peripheral sensory organs is first processed in the primary olfactory neuropil of the brain, the antennal lobe, whereafter the information is relayed to secondary brain neuropils such as the mushroom body (Hansson, 1999). Both antennal lobes and the mushroom body input region, the calyx, are known to fluctuate in size and may grow larger as they process an increasing amount of information (Anton et al., 2016; van Dijk et al., 2017; Durst et al., 1994; Eriksson et al., 2019; Gronenberg et al., 1996; Heisenberg et al., 1995; Jones et al., 2013; Kühn‐Bühlmann & Wehner, 2006; Maleszka et al., 2009; Montgomery et al., 2016; Snell‐Rood et al., 2009; Withers et al., 1993, 2008). Although it has been demonstrated that olfactory long‐term memory in honeybee may only affect the density of calycal microglomeruli and not the global calyx size (Hourcade et al., 2010), larger brains are generally assumed to have increased capability for information processing, and sensory neuropil size is considered an indication for how important the related sensory system is to an insect (Gronenberg & Hölldobler, 1999; Heinze & Reppert, 2012; Montgomery & Ott, 2015; Stöckl et al., 2016). This means that relative neuropil size could be indicative of how important a sensory system is to an individual, dependent on the specific ecological setting of its present environment.

As adult insects have a fully developed neural infrastructure for sensory processing and may have conflicting selection pressures regarding sensory neuropil size during dormancy, they are well‐suited for studying the effects of diapause dormancy on brain tissue investment strategies. *Polygonia c‐album* is a butterfly with (a) a facultatively induced adult diapause where the decision of entering diapause is made before adult eclosion (Nylin, 1989), (b) a documented high potential for structural plasticity of olfactory neuropils (van Dijk et al., 2017; Eriksson et al., 2019), and (c) a life cycle which is easily manipulated under laboratory conditions. We approached the question of when investments into the olfactory system are made by measuring the volumes of mushroom body calyces and antennal lobes at five time points before, during, and after diapause dormancy of female *P. c‐album* butterflies. As the pre‐ and postdiapause periods naturally are characterized by very different ecological needs, these differences may be reflected in the functional drivers of brain plasticity. Therefore, we also discuss the nature of investment strategies observed before and after diapause from a perspective of experience dependency. Our results indicate differences in the mechanisms governing structural plasticity during the different life stages and show a clear pattern of when investments into olfactory neuropils are made.

## 2 MATERIALS AND METHODS

### 2.1 Species and rearing

**FIGURE 1:**
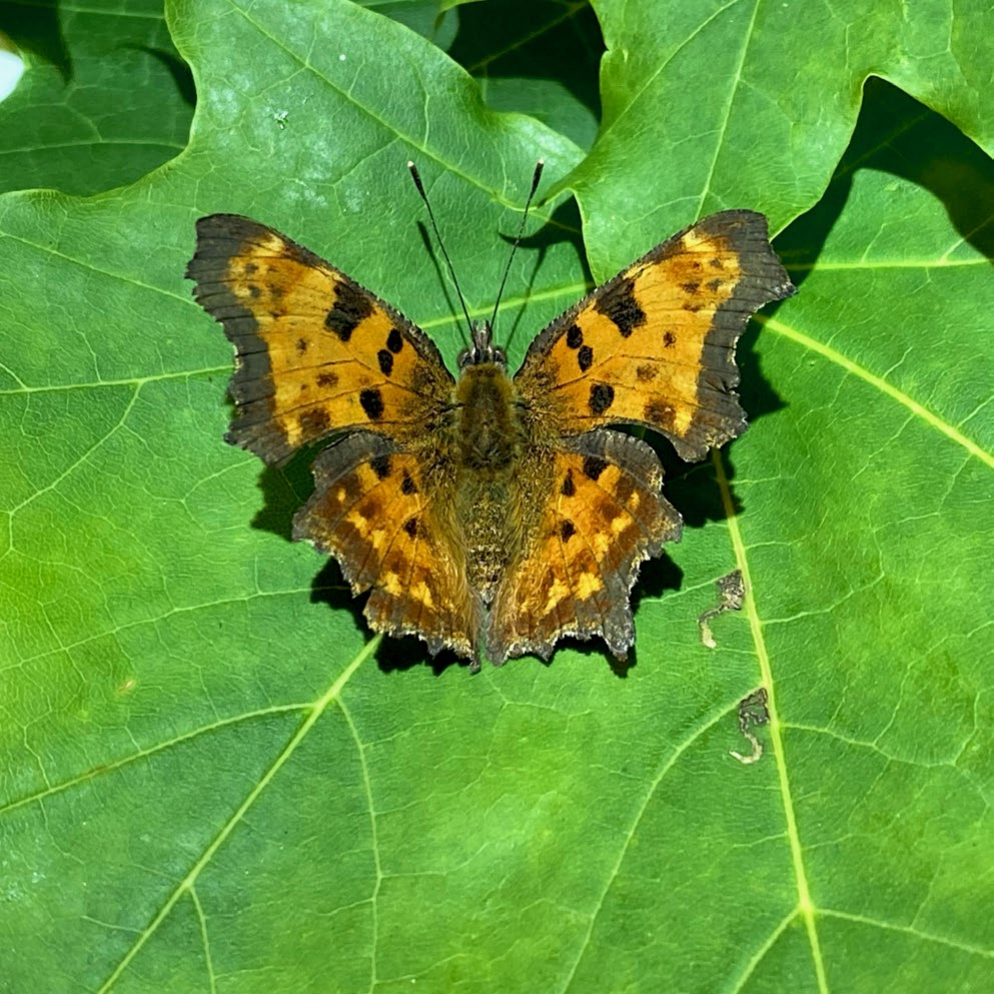
*Polygonia c‐album*, female on maple leaf

The Comma butterfly *P. c‐album* (Linné, 1758) (Figure 1) has a large distribution area, spanning from Great Britain and northern Africa in the west, to Japan in the east. It is a facultatively multivoltine species, having more than one generation per year in areas where the summer season is sufficiently long, and undergo diapause as imago. Uniform daylength, especially if shorter than 13 hr, favors the developmental path leading to diapause readiness, while increasingly longer days (e.g., L:D 12:12→22:2) experienced during larval development favors the path leading to directly reproducing adults, especially when accompanied with increasingly higher temperatures (e.g., 17°C→23°C).

*Polygonia c‐album* is considered a polyspecialist with regard to host plant utilization, and olfaction is likely to play an important role during the search for suitable oviposition sites as mated females are known to show attraction toward host plants based solely on olfactory cues (Schäpers et al., 2015).

A cohort of *P. c‐album* females, derived from a laboratory population originating in the Norfolk region of the United Kingdom and provided by the company Worldwide Butterflies, was used in this study. Larvae from approximately 30 egg‐laying females were reared on fresh cuts of stinging nettles *Urtica dioica* under a light and temperature regime promoting the development of diapausing adults (12:12 L:D, 17°C). Pupae were removed from their pupation sites two days after pupation, sexed by inspection of the genital slits, and placed in paper‐lined cups covered with mesh nets until adult eclosion.

### 2.2 Conditions during treatment

One‐day‐old adults were transferred to mesh net cages (50 cm*50 cm*50 cm) in a secluded room with a neutral olfactory environment. The room was under a 6:18 L:D cycle, and a dynamic temperature cycle peaking at 27°C during the end of the light period, and dropping down to 20°C during the dark period. Cages were equipped with feeding stations consisting of a plastic cup with a dish sponge and freshly mixed 25% sugar water. Feeding stations were regularly exchanged in order to prevent growth of algae and fermentation of the sugar as to avoid nonstandardized variations in gustatory and olfactory conditions during the time spent in flight cages.

Butterflies were kept in the flight cages for 14 days before the diapause dormancy climate conditions were initiated and again for two weeks after the dormancy conditions were terminated. Throughout the period when dormancy conditions were active, the butterflies were kept in plastic cups covered with mesh nets (secured with rubber bands) and placed in a cardboard box which in turn was kept in a sheltered area on the roof of the Zoology Department of Stockholm University during the winter of 2018–2019.

All butterflies were placed on the underside of the mesh net, inside the cups, as this position seems to increase diapause survival rates (Christer Wiklund, Stockholm University, 2018.07, personal communication). The animals were regularly checked on during the dormancy period, and individuals which had dropped off from the net were gently put back into their original position if still alive at the time of discovery.

### 2.3 Sampling

Butterflies were sampled at five different time points: (a) 1 day after adult eclosion, (b) immediately before the onset of dormancy conditions, at 14 days after adult eclosion, (c) during the midstage of the diapause dormancy period, approximately two months after dormancy conditions were initiated, (d) at the end of diapause dormancy, approximately four months after dormancy conditions were initiated, and (e) two weeks after dormancy conditions were terminated and butterflies had been returned to indoor flight cages.

It should be noted that *P. c‐album* butterflies generally take flight within minutes and start feeding within hours after diapause dormancy climate conditions are terminated, and are ready to mate within a few days up to one week (M. E., unpublished observation).

### 2.4 Dissection and sample preparation

Butterflies were decapitated using microscissors, and their heads were fixated overnight at 4°C in a 4% paraformaldehyde (Sigma‐Aldrich, Steinheim, Germany) solution. Heads were washed in phosphate‐buffered saline (Sigma‐Aldrich, Steinheim, Germany) containing 2.5% Triton‐X (Sigma‐Aldrich, Steinheim, Germany) (PBStx) 4 × 15 min before the brains were dissected out. Brain samples were incubated in a 1:20 solution of primary antibody targeting synapsin (3C11 anti‐SYNORF1; Developmental Studies Hybridoma Bank) and PBStx with 0.5% bovine serum albumin (BSA; Sigma‐Aldrich, St. Louis, MO, USA) over 3 days at 4°C on a shaker at low setting. Samples were then washed 5 × 1 hr before incubation in secondary antibody (Alexa Fluor 488 [Life technologies] 1:500 in PBStx 0.5% BSA) for 3 days at the same conditions as for primary incubation. Stained samples were washed 4 × 1hr in PBStx and 1 × 1hr in PBS, and treated for optical clearing in Omnipaque (GE Healthcare AS), firstly in a 1:1 solution of Omnipaque:PBS for 24 hr and then stored in pure Omnipaque for a minimum of 24 hr. As clearing in Omnipaque eliminates the need for sample dehydration in ethanol and usage of methyl salicylate, the tissue shrinking associated with such protocols (Bucher et al., 2000; Smolla et al., 2014) is avoided, allowing for more accurate volume measurements. Omnipaque is an odorless and benign liquid commonly used in clinical treatments, making it safe and easy to work with.

### 2.5 Confocal scanning and reconstruction

Cleared samples were whole‐mounted in Omnipaque on glass slides with custom‐made 0.5 mm metal spacers and were optically sectioned using a Zeiss LSM 780 META (Zeiss) scanning confocal laser microscope. Images with a resolution of 1,024 × 1,024 pixels were obtained with a 10× air objective. Each section had a thickness of approximately 3 μm, resulting in image stacks of about 100 sections per sample.

3D reconstruction and extraction of volumetric measurements (in μm^3^) from antennal lobes, mushroom body calyces, and whole central brain regions (optic lobes were excluded from analysis) were performed by using the native segmentation, volume rendering, and surface reconstruction tools in the Thermo Scientific^™^ AMIRA^™^ (v. 2019.3) image processing software.

Samples that were physically damaged during technical processing were excluded from analysis, as were those that did not render high‐quality images during confocal scanning, or had physical abnormalities such as divergent number of calycal cups. In cases where only one of a paired neuropil was discarded for the previously stated reasons, the intact neuropil volume was duplicated as to achieve total neuropil volumes comparable to fully intact samples. As paired neuropils are known to be symmetrical between hemispheres (Eriksson et al., 2019; Galizia et al., 1998; el Jundi et al., 2009), both neuropils were discarded in cases where the volume difference was greater than 10% within a pair. The latter phenomenon is to be expected in cases where the neurolemma was ruptured before samples were fully fixated, allowing damaged tissues to expand post mortem.

### 2.6 Data processing and statistics

Relative volumes of mushroom body calyces and antennal lobes were obtained by dividing absolute neuropil volumes over whole central brain volumes (including antennal lobes and calyces, but excluding the optic lobes) for each individual. As relative volumes identify the proportion of the central brain that is occupied by a specific neuropil, it highlights disproportionate volume fluctuations which otherwise could be obscured by global changes in brain volume, and it also negates statistical noise introduced by global size differences between individuals and allows comparisons of results between studies employing somewhat different laboratory protocols.

Statistical analyses were performed using GraphPad Prism version 8.4.2 for MacOS (GraphPad Software; www.graphpad.com). Initial analyses confirmed that all assumptions for ANOVA were fulfilled. One outlier was detected in the relative calyx volume measurements among newly eclosed butterflies and was subsequently removed. Changes in absolute and relative volume measurements over time were analyzed by ordinary one‐way ANOVA followed by Tukey's multiple comparisons test.

### 2.7 Comparison with butterflies of a nondiapausing conspecifics

Data on central brain and olfactory neuropil volumes of female *P. c‐album* butterflies from a nondiapausing generation were recently published by our research group (van Dijk et al., 2017). All females used in the present study are members of the same laboratory contained population as those of the previous study, although by several generations their successors. Due to differences in sample preparation (clearing with Omnipaque vs. methyl salicylate), absolute brain volumes cannot be compared between studies, but as the tissue shrinkage caused by traditional clearing protocols is assumed to be uniform, comparisons of relative neuropil volumes are possible.

No permits were needed for this study on invertebrates performed in Sweden.

## 3 RESULTS

By reconstructing the central brain and olfactory neuropils as digital 3D models from images obtained with a confocal laser scanning microscope, we measured the volumes of these brain regions from samples collected at five different time points before, during, and after the diapause dormancy period of unmated female *P. c‐album* butterflies. (See figure in van Dijk et al. (2017) for illustration of brain and reconstruction.)

### 3.1 Central brain

**FIGURE 2:**
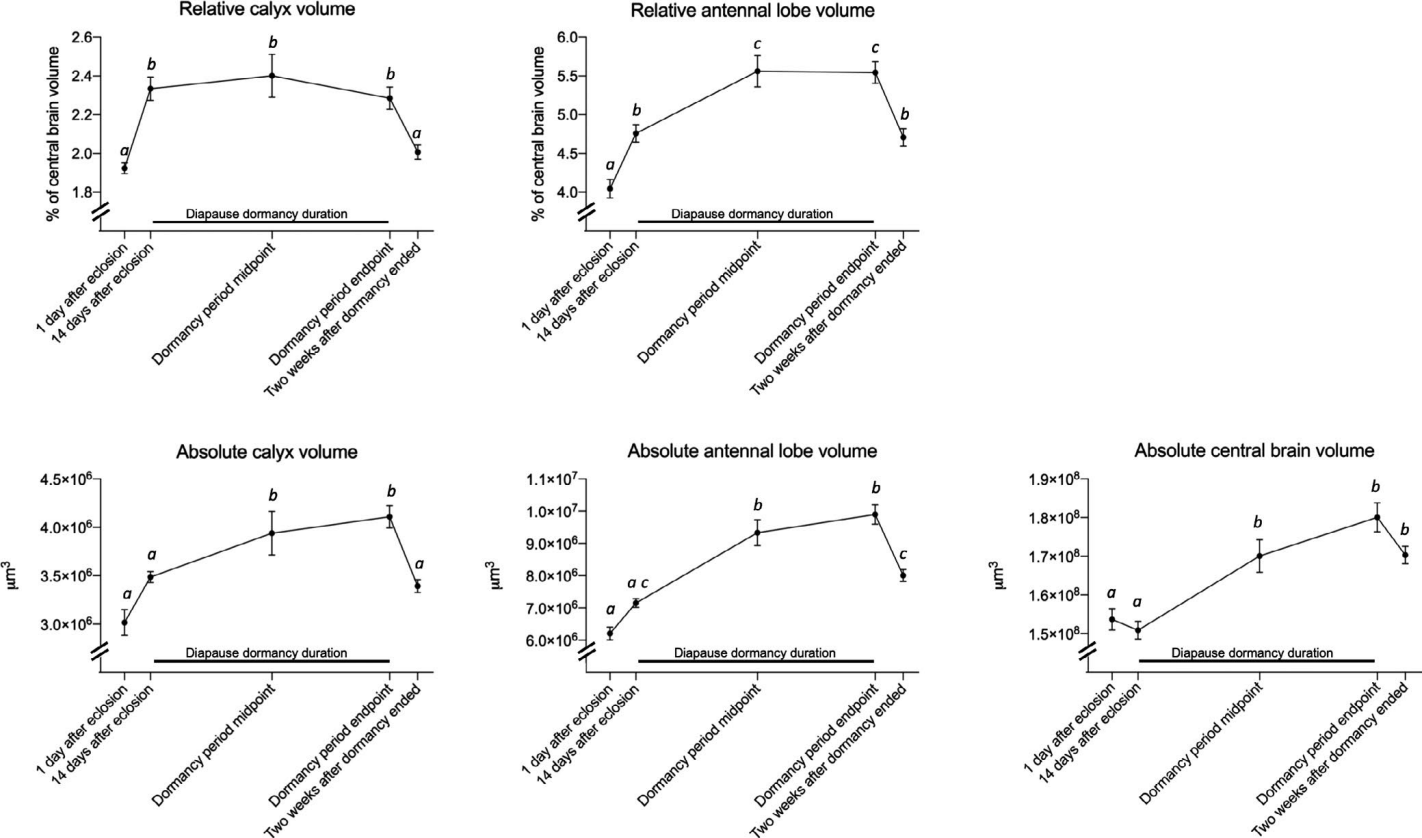
Average volumes of antennal lobes, mushroom body calyces, and central brain regions of *Polygonia c‐album* butterflies at different time points before, during, and after diapause dormancy. Diapause dormancy climate conditions were initiated 14 days after adult eclosion and lasted approximately four months. Top row shows relative neuropil size as a percentage of total central brain volume, while the bottom row shows absolute volumes as measured in μm^3^. Error bars indicate SE, and different letters indicate statistically significant differences in volume between sample points

The central brain region shows no volumetric change during the first 14 days after eclosion, but increases in volume by about 13% during the first half of the dormancy period (*p* < .0005; Figure 2; Table 1). There are no further statistically significant changes in central brain volumes throughout the rest of the experiment.

**TABLE 1:** Absolute and relative volumes of measured brain regions and changes in volume measurements over time

	Central brain Volume	Abs. calyx Volume	Abs. antennal lobe Volume	Rel. calyx Volume	Rel. antennal lobe Volume
**a)**					
1 day after eclosion	1.5 × 10^8^ mm^3^ (*SE* = 2.8 × 10^6^ *n* = 15)	3.0 × 10^6^ mm^3^ (*SE* = 1.3 × 10^5^ *n* = 9)	6.2 × 10^6^ mm^3^ (*SE* = 2.0 × 10^5^ *n* = 15)	1.90% (*SE* = 2.8 × 10^−4^ *n* = 8)	4.00% (*SE* = 1.2 × 10^−3^ *n* = 15)
14 days after eclosion	1.5 × 10^8^ mm^3^ (*SE* = 2.3 × 10^6^ *n* = 15)	3.5 × 10^6^ mm^3^ (*SE* = 5.8 × 10^4^ *n* = 11)	7.2 × 10^6^ mm^3^ (*SE* = 1.4 × 10^5^ *n* = 12)	2.30% (*SE* = 6.0 × 10^−4^ *n* = 11)	4.80% (*SE* = 1.1 × 10^−3^ *n* = 12)
After first half of the dormancy period	1.7 × 10 mm^3^ (*SE* = 4.2 × 10^6^ *n* = 11)	4.1 × 10^6^ mm^3^ (*SE* = 2.4 × 10^5^ *n* = 11	9.3 × 10^6^ mm^3^ (*SE* = 4.0 × 10^5^ *n* = 12)	2.40% (*SE* = 1.1 × 10^−3^ *n* = 10)	5.60% (*SE* = 2.0 × 10^−3^ *n* = 11)
After second half of the dormancy period	1.8 × 10 mm^3^ (*SE* = 3.7 × 10^6^ *n* = 9)	4.1 × 10^6^ mm^3^ (*SE* = 1.2 × 10^5^ *n* = 9)	9.9 × 10^6^ mm^3^ (*SE* = 3.0 × 10^5^ *n* = 11)	2.30% (*SE* = 5.8 × 10^−4^ *n* = 9)	5.50% (*SE* = 1.4 × 10^−3^ *n* = 9)
2 weeks after the dormancy period had ended	1.7 × 10 mm^3^ (*SE* = 2.3 × 10^6^ *n* = 14)	3.4 × 10^6^ mm^3^ (*SE* = 6.7 × 10^4^ *n* = 12)	8.0 × 10^6^ mm^3^ (*SE* = 1.9 × 10^5^ *n* = 14)	2.00% (*SE* = 3.7 × 10^−4^ *n* = 12)	4.70% (*SE* = 1.1 × 10^−3^ *n* = 14)
**b)**					
1 day after eclosion versus. 14 days after eclosion	−1.80% *ns*	+15.60% *ns*	+15.20% *ns*	+21.40% (*F*_4,45_ = 10; adj. *p* = .001)	+17.70% (*F*_4,56_ = 21; adj. *p* < .005)
14 days after eclosion versus. After first half of the dormancy period	+12.70% (*F*_4,57_ = 16; adj. *p* < .0005)	+16.40% (*F*_4,47_ = 11; adj. *p* < .05)	+30.50% (*F*_4,59_ = 36 adj. *p* < .0001)	+2.90% *ns*	+16.80% (*F*_4,56_ = 21; adj. *p* < .005)
After first half of the dormancy period versus. After second half of the dormancy period	+5.90% *ns*	+1.20% *ns*	+6.10% *ns*	−4.80% *ns*	−0.30% *ns*
After second half of the dormancy period versus. 2 weeks after the dormancy period had ended	−5.40% *ns*	−17.40% (*F*_4,47_ = 11; adj. *p* < .01)	−19.40% (*F*_4,59_ = 36; adj. *p* < .0001)	−12.20% (*F*_4,45_ = 10; adj. *p* < .05)	−15.10% (*F*_4,56_ = 21; adj. *p* < .005)

### 3.2 Mushroom body calyx

Relative mushroom body calyx volumes, as compared to central brain volumes, increase by 21% during the first 14 days after eclosion (*p* < .01; Figure 2; Table 1). There is a weak trend of increasing absolute volumes during these 14 days, but absolute volumes increase significantly only during the first half of the dormancy period (*p* < .05). There are no significant changes in mushroom body calyx volumes during the second half of the dormancy period. During the two weeks after dormancy conditions were terminated, relative calyx volumes decrease by 12% (*p* < .05) and absolute volumes decrease by 17% (*p* < .01).

### 3.3 Antennal lobes

Relative antennal lobe volumes, as compared to central brain volumes, increase by 18% during the first 14 days after eclosion (*p* < .005) and again increase by 17% during the first half of the dormancy period (*p* < .005; Figure 2; Table 1). There is a trend of increasing absolute volumes during the first 14 days after eclosion, but absolute volumes increase significantly only during the first half of the dormancy period (*p* < .0001). There are no significant changes in antennal lobe volumes during the second half of the dormancy period. During the two weeks after dormancy conditions were terminated, relative antennal lobe volumes decrease by 15% (*p* < .005) and absolute volumes decrease by 19% (*p* < .0001).

### 3.4 Comparison with butterflies of a nondiapausing generation

Relative olfactory neuropil volumes of female butterflies from a nondiapausing generation were recently quantified and published by our research group in a previous study (van Dijk et al., 2017). The same captive‐bred *P. c‐album* stock population was used in both studies, although the present study used individuals sampled from a later generation. A comparison of the measurements observed in the two studies reveals a large difference in relative neuropil volumes between diapausing and nondiapausing generations. Firstly, we see that females destined for diapause eclose with approximately 35% smaller calyces and approximately 50% smaller antennal lobes than nondiapausing females do (relative to central brain volumes). Secondly, as we compare the 14 days old females in the present study with females of similar age and experience in the van Dijk study (*mated only* treatment) we see that this size difference largely is maintained throughout early adulthood.

### 3.5 Summary

A temporal pattern of brain development emerges when comparing the volumes of different brain regions between sample points. Increases in relative neuropil volumes mainly occur during the first 14 days after eclosion. The only time period in which absolute volumes of all and any brain regions increase significantly is during the first half of the dormancy period. No changes are observed during the second half of the dormancy period, indicating that the diapause dormancy period is divided into different phases distinguished by absence or presence of significant brain growth. Both absolute and relative volumes of the olfactory neuropils decrease substantially during the two weeks after dormancy conditions were terminated, despite butterflies being kept at the same conditions as before dormancy conditions were initiated. In addition to this pattern, we can also see that the relative volumes of calyces and antennal lobes in the present study are remarkably smaller, both at eclosion and at two weeks of age, compared with those of females from a nondiapausing generation.

## 4 DISCUSSION

Our results show clear temporal patterns as to when investments into antennal lobes and mushroom body calyces are made in relation to the adult diapause dormancy period of the butterfly *P. c‐album*. Relative volumes of both neuropils, as compared to the whole central brain region, *increase* significantly during the first 14 days after eclosion and *decrease* significantly during the two weeks after the dormancy period had ended. Importantly, the central brain region does not change significantly in volume during the pre and postdormancy periods, thus excluding artifacts of global brain growth, osmotic swelling, and general neural regression as causes for the observed changes in relative olfactory neuropil volumes during these time periods (Lehmann et al., 2017). The diapause dormancy period itself appear to contain at least two distinct phases as absolute volumes of both olfactory neuropils and the central brain region increase significantly during the first half, but do not change during the second half of the dormancy period.

When changes in brain volumes are observed over time, they are often categorized as either experience‐dependent or experience‐*in*dependent. Experience‐dependent investments occur as a result of various individual experiences and are expected to improve proficiency in overcoming challenges encountered in the present environment. Experience‐independent investments, on the other hand, can be described as experience‐*expectant* if they allow the animal to be better prepared for tackling future challenges (Fahrbach et al., 1998). The latter type of investments would be driven by preprogrammed ontogenetic protocols and could give insights regarding the challenges which are to be expected at different time points in the life of an insect of a specific species, population, or developmental pathway.

In order to determine whether the structural plasticity we see in the present study definitely belongs to one of these categories or the other, further studies would be needed. Treatments which force decreased levels of natural stimulation, for example, by immobilization and nondestructive blockage of odor detection (Eriksson et al., 2019), and those which allow increased stimulation by enriched environments may further our understanding in this area. However, conclusions from earlier studies allow us to evaluate the likelihood of drivers for the presently observed plasticity to belong in either category.

The mushroom body is important for a variety of memory functions and learning abilities (de Belle & Heisenberg, 1994; Fahrbach, 2006; Farris & Van Dyke, 2015; Giurfa, 2013; Heisenberg, 1998, 2003; Heisenberg et al., 1985; Zars, 2000; Zars et al., 2000; Zhang et al., 2013), and the calyx is known to expand volumetrically after activities such as general foraging and challenging cases of host plant search (van Dijk et al., 2017; Durst et al., 1994; Jones et al., 2013; Maleszka et al., 2009; Montgomery et al., 2016; Snell‐Rood et al., 2009). Based on this, it is not unlikely to assume that there could be a causal link between general experience‐dependent increases in calyx volume and behaviors related to spatial navigation, learning, and similar cognitive processes. In nondiapausing *P. c‐album* females, it has previously been shown that mushroom body calyx growth can be attributed partly to experience‐dependent processes (van Dijk et al., 2017) and partly to experience‐independent processes (Eriksson et al., 2019). In the former case, relative calyx volumes were shown to increase with increasing complexity of plant compositions experienced after mating. Similarly, a positive correlation between calyx size and challenging plant environments has been observed among reproductive *Pieris rapae* butterflies (Snell‐Rood et al., 2009), and a connection between calyx volume and learning was suggested.

The butterflies in the present study were kept in a neutral environment with no plants, low exposure to ecologically relevant olfactory cues, little space for flight navigation, and with very limited opportunity for learning. Relative calyx volumes also decreased during the two weeks after the dormancy period had ended, despite butterflies being kept under the same conditions as during the first 14 days after eclosion. With this in mind, it seems likely that the presently observed increase in relative calyx volume prior to the dormancy period mostly would fall in the category of experience‐expectant growth.

The antennal lobe is a site of convergence for axons of olfactory receptor neurons (Galizia & Rössler, 2010; Vosshall et al., 2000), and the received signals may undergo significant modulation within the neuropil itself before being transferred to upstream brain regions (Carlsson et al., 2010, 2013; Galizia & Rössler, 2010; Kloppenburg & Mercer, 2008; Krofczik et al., 2009; Kuebler et al., 2012; Sachse & Galizia, 2002). Although less commonly reported than for mushroom bodies, both age‐dependent growth (Gronenberg et al., 1996; Montgomery et al., 2016) and experience‐dependent growth (Eriksson et al., 2019; Jones et al., 2013) of whole antennal lobes have been observed before, as having age‐ and experience‐dependent plasticity of individual antennal lobe glomeruli (Anton et al., 2016; Arenas et al., 2012; Brown et al., 2004, 2018; Devaud et al., 2001, 2003; Guerrieri et al., 2012; Huetteroth & Schachtner, 2005; Sachse et al., 2007; Sigg et al., 1997; Winnington et al., 1996; Withers et al., 1993). It was recently shown that there is no age‐dependent increase in antennal lobe volumes of nondiapausing *P. c‐album* females, but that volumes may increase substantially in the presence of olfactory input (Eriksson et al., 2019). As with calyx volume, considering the sparse nature of the provided odor environment and decreasing antennal lobe volumes after the dormancy period had ended, it is likely that the presently observed increase in relative antennal lobe volume prior to the dormancy period mostly would fall into the category of experience‐expectant growth.

Despite an apparent pressure for energy conservation, we see substantial investments into presumed experience‐expectant growth of olfactory‐related neuropils prior to the diapause dormancy period. While this can give an indication of the importance of having a well‐developed olfactory system with high capacity for olfactory processing ready to be used as the mating season starts, it does not on its own allow broader conclusions regarding energy conservation in a more general diapause‐related perspective. Substantial differences are, however, revealed when comparing the relative neuropil volumes observed in the present study with those of females from a nondiapausing generation (van Dijk et al., 2017). Calyces of newly eclosed nondiapausing butterflies are larger by about a third and antennal lobes are approximately twice as large as those in the present study, relative to whole central brain volumes. The differences in relative neuropil volumes between generations are similar also between the 14‐day‐old females in the present study and butterflies of comparable age and experience in the van Dijk study (van Dijk et al., 2017). The consistent differences indicate that prediapause and nondiapausing butterflies could have different investment strategies regarding brain development, both before and after adult eclosure. In this context, it is important to note that there is a relatively tight connection between wing coloration and developmental pathway (the diapause form is associated with darker wing colors; Nylin, 1992) and that pathway decisions are induced during larval development (Nylin, 1989). This means that, in contrast to even some closely related butterflies such as *Aglais urticae* (Wiklund et al., 2019), it is fairly easy to determine to which developmental pathway an individual *P. c‐album* butterfly belongs by visual inspection. As the needs connected to behavior and ecology undoubtedly differ between seasons, differences in resource investment strategies between butterflies that do and do not undergo diapause prior to the season of reproduction are not entirely unexpected. Considering the metabolic cost attached to maintenance of neural tissues, we argue that the substantially reduced size of olfactory neuropils found in diapause‐ready butterflies, compared with nondiapausing counterparts, could be viewed as a measure of energy conservation in anticipation of expected conditions during the coming winter season.

Relative olfactory neuropil volumes decreased substantially during the two weeks after the dormancy period had ended, despite butterflies being kept under the same conditions as before the dormancy period started. The lack of growth aligns well with expectations for experience‐dependent investment strategies under low‐stimuli conditions, but reductions in neuropil volume are scarcely reported for insects. Such observations, when reported, are generally limited to smaller substructures such as the lobula and medulla of the optic lobes (Gronenberg et al., 1996; Julian & Gronenberg, 2002), and individual antennal lobe glomeruli (Devaud et al., 2001, 2003; Sachse et al., 2007; Winnington et al., 1996; Withers et al., 1993). A common theme for neuropil reductions in insects appears to be that they mainly occur after a period of growth and generally do not cause volumes to decrease below levels observed for very young adults. This pattern appears to hold true also for some birds where seasonal fluctuations in volumes of a brain region associated with spatial memory and foraging, the hippocampus, have been observed (Sherry & Hoshooley, 2010). Hypothetically, there could be mechanisms in place which prohibit shrinkage below a certain threshold level in order to protect basic functionality of the neuropil. Such a mechanism could explain why we see volumetric reductions in postdiapause butterflies but not in (comparatively young) nondiapausing butterflies deprived of olfactory stimulation (van Dijk et al., 2017; Eriksson et al., 2019), but this is purely speculative. The occurrence of a shift from growth to shrinkage is, however, very clear in this study and, as butterflies of this species become reproductively active soon after spring emergence, this shift in brain development coincides with a hard shift from premating season to mating season. It should also be noted that as there is no significant change in central brain volume during this time, the reduction in olfactory neuropil volumes is highly salient. The stark contrast in ecological profiles between the two life stages supports the assumption of different investment strategies, as the demands and needs of each life stage are vastly different. Therefore, we argue that the difference in mushroom body calyx and antennal lobe development patterns observed before and after the diapause dormancy period most parsimoniously can be explained with a shift in investment strategies. Thus, while the increase in size of the olfactory structures before diapause likely is experience‐expectant, the postdiapause decrease may be dependent on experience (or rather lack thereof under the present experimental conditions). In theory, the shift from a preprogrammed to an experience‐dependent developmental regime once the mating season starts could represent a way of neutralizing the conflict between the need for minimized metabolic cost during diapause and high capacity for sensory processing during the season of reproduction.

## 5 CONCLUSION

The smaller volume of olfactory neuropils observed in diapause‐ready butterflies, compared with nondiapausing individuals and coupled with the expected metabolic cost of neural tissue maintenance, indicate the presence of a tissue investment strategy adapted to promote low energy consumption during diapause. The opposite direction of changes in olfactory neuropil volumes before and after diapause indicates that brain development could be managed by different investment strategies at different life stages. Our results suggest that structural neuropil plasticity would be governed by experience‐expectant processes before diapause and by experience‐dependent processes after diapause is broken and the mating season has begun. Taken together, these observations could be indicative of evolutionary adaptations that mitigate the trade‐off between dormancy survival and reproductive fitness.

## CONFLICT OF INTEREST

The authors declare to have no conflict of interest.

## AUTHOR CONTRIBUTION

**Maertha Eriksson:** Conceptualization (supporting); Data curation (lead); Formal analysis (lead); Investigation (equal); Methodology (equal); Visualization (lead); Writing‐original draft (lead); Writing‐review & editing (lead). **Niklas Janz:** Conceptualization (supporting); Investigation (equal); Methodology (equal). **Sören Nylin:** Conceptualization (supporting); Funding acquisition (lead); Investigation (equal); Methodology (equal). **Mikael Carlsson:** Conceptualization (lead); Data curation (supporting); Formal analysis (supporting); Investigation (equal); Methodology (equal); Project administration (lead); Visualization (supporting); Writing‐original draft (supporting); Writing‐review & editing (supporting).

## Data Availability

Volumetric data are available from the Dryad Digital Repository at: https://doi.org/10.5061/dryad.cjsxksn4s
